# Development of Alkali-Activated Porous Concrete Composition from Slag Waste

**DOI:** 10.3390/ma16041360

**Published:** 2023-02-06

**Authors:** Gintautas Tamošaitis, Danute Vaičiukynienė, Tomas Jaskaudas, Jurate Mockiene, Darius Pupeikis

**Affiliations:** Faculty of Architecture and Civil Engineering, Kaunas University of Technology, Studentų st. 48, 51367 Kaunas, Lithuania

**Keywords:** alkali-activated slag, phosphogypsum, porous concrete, thermal conductivity

## Abstract

**Highlights:**

The precursor of alkali activated slag was made from slag and 0, 3, 5, 7 and 10% phosphogypsum.

Phosphogypsum and H_2_O_2_ led to positive changes of porous alkali-activated slag concrete.

The recommended content of phosphogypsum is 3–5% in alkali-activated slag system.

Porous concrete had a compressive strength of 2.12–7.95 MPa, a density of 830 kg/m^3^–1142 kg/m^3^, and a thermal conductivity of 0.0985–0.2618 W/(m·K).

This porous concrete is recommended for the production of low-strength insulation blocks.

**Abstract:**

In this paper, a porous alkali-activated slag concrete was developed that can be used in the construction sector as a sustainable building material and potentially as an alternative to the aerated concrete products currently on the market. Ferrous slag from the metallurgical industry (Finland) and phosphogypsum from a fertilizer plant (Lithuania) were used as precursors in alkali-activated systems. The addition of hydrogen peroxide and phosphogypsum led to positive changes in the final properties of the test material. Porous concrete based on alkali-activated slag was analyzed by X-ray diffraction (XRD), Fourier transform infrared (FTIR) and scanning electron microscopy (SEM) methods. The compressive strength, density, thermal conductivity and porosity of the hardened specimens were evaluated as well. Research is being conducted with the material in question to create a cheap, particularly low-energy demanding building material. This material must have suitable mechanical properties for the structure and, at the same time, suitable thermal conductivity properties. It was determined that this porous concrete had compressive strength in the range of 2.12–7.95 MPa, density from 830 kg/m^3^ to 1142 kg/m^3^, and thermal conductivity in the range of 0.0985–0.2618 W/(m·K). The results indicate that the recommended content of phosphogypsum in alkali-activated material is 3–5% due to the optimal distribution of the mechanical and thermal properties and the conductivity. Alkali-activated slag and phosphogypsum material can be used in the manufacture of low-strength insulation blocks and to protect structures from the effects of high temperatures.

## 1. Introduction

Alkali-activated materials (AAM) are actively analyzed and described by today’s researchers as the relevance of their application becomes more and more important in today’s world. According to the life cycle assessment of the materials, the global warming potential of alkali-activated cementitious material is ~55–75% lower than that of cementitious materials with ordinary Portland cement. The CO_2_ emissions released into the atmosphere during the manufacturing process of alkali-activated concrete are 20% of the normal CO_2_ emissions of Portland cement concrete [1]. So, alkali-activated materials (binders) are one of the fastest-growing alternatives to replace conventional Portland cement due to the use of industrial waste.

Currently, the potential applications of metallurgical slag in alkali-activated systems are widely studied and analyzed in the scientific literature. Sun et al. [2] revealed that alkali-activated ground granulated blast furnace slag (GGBFS) resulted in a higher amount of microcracks and anhydrate particles, which could be related to the lower compressive strength than ordinary Portland cement. Rashad et al. [3] found a positive effect of quartz powder when used in alkali-activated slag specimens. Aziz et al. [4] determined that alkali-activated GGBFS specimens had high compressive strength (168.7 MPa) after 28 days of hydration, and this development of compressive strength could be related to the formation of tobermorite gel and calcite (CaCO_3_) in the mineral composition of specimens. The blends based on slag with the addition of phosphogypsum could be used in the alkali activation process due to the positive influence of phosphogypsum on the mechanical properties and the hydration process [5]. Gijbels et al. [6] found that the addition of phosphogypsum can lead to higher polymerization density and higher compressive strength values. Therefore, the incorporation of phosphogypsum into the alkali-activated slag systems is a good method for full utilization.

Due to the high concentration of alkaline activator, porous specimens can be formed quite easily. Similarities can be observed between this process and the technology of aerated concrete, in which the foaming agent is mixed with a binder, and a product with a closed network of pores is formed. The most used foaming agents in practice are aluminum powder, which releases hydrogen gas during the reaction, hydrogen peroxide, which releases oxygen during the reaction, and sodium carbonate, which releases carbon dioxide. This sequence of the activation process ensures the formation of a closed pore network of the material. High porous specimens reached 3.3 MPa of compressive strength with 0.2% aluminum powder and 3.7 MPa of compressive strength using 2.0% H_2_O_2_ [7]. Porous cementitious materials are often used as thermal barriers, preventing thermal energy from escaping easily from a building. Pore size, distribution, density of the material and type of filler are the most important factors that determine the thermal properties of the material. Şahin et al. [8] prepared alkali-activated slag specimens with bulk density ranging from 516 kg/m^3^ to 1199 kg/m^3^, compressive strength of 0.5–30.0 MPa, and thermal conductivity from 0.117 to 0.206 W/m·K. Hajimohammadi et al. [9] argued that the main factor of thermal conductivity is the homogeneity of pore distribution, which is closely related to the increase in strength. The by-product perlite was used to produce alkali-activated porous materials. This type of material had a low thermal conductivity (0.03 W/m·K), a density of 290 kg/m^3^, and a compressive strength of 0.78 MPa [10]. Esmaily et al. [11] stated that compressive strength is largely affected by the structure of pores. In cellular alkali-activated slag, the structure of optimal pores was created by optimizing the sodium silicate modulus. The physical and mechanical properties of a porous material depend greatly on how the pores of different sizes are distributed in the material. Materials with the same total porosity can have completely different properties due to the small number of large pores or the larger number of small pores [12,13].

The aim of this study is to determine the influence of phosphogypsum addition on the density, compressive strength, mineral composition, pore distribution, thermal conductivity and microstructure of porous alkali-activated slag.

## 2. Experimental Procedures

The chemical composition of the ferrous slag and phosphogypsum was evaluated by XRF analysis. A fluorescence spectrometer was used for this analysis [14]. The mineral compositions of the materials (slag and phosphogypsum) and the hardened porous alkaline-activated slag were evaluated by XRD analysis. An X-ray diffractometer was used to determine the mineral composition [15]. The particle size distribution of the slag and phosphogypsum was determined using a laser particle size analyzer [16]. Microscopic analysis, such as the scanning electron microscopy (SEM) of the starting materials (ferrous slag and phosphogypsum) and the hardened porous alkaline-activated slag, was performed using a high-resolution scanning electron microscope [17]. The compressive strength was determined after 3 and 28 days of hydration. A hydraulic press was used to determine the compressive strength. The density of the porous AAM was determined according to EN 12390-7, and the compressive strength of the specimens according to EN 196-1. For the determination of thermal conductivity, the specimens were prepared according to the standard LST EN 12667 (2002). In this case, a heat flux meter was used [18].

**Figure 1:**
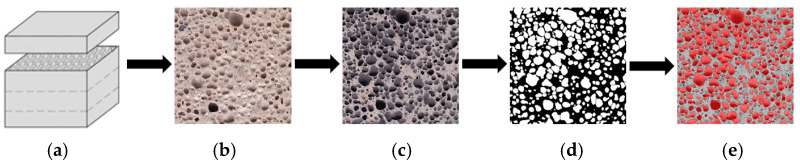
Diagram for determining the cross-sectional distribution of air pores. (**a**) Original cross-section; (**b**) scanned specimen surface; (**c**) paint applied to specimen surface; (**d**) scanned image converted to binary image to calculate pore size and area; (**e**) air pores highlighted in cross section.

Air voids and their formation are one of the most important parameters in alkali-activated, low-density, hardened structures. The ASTM C457 standard describes some of the procedures for this task. In this paper, a modified alternative to the surface and void calculation method (Figure 1) is used, which involves scanning the surface of a ground and further prepared specimen.

This alternative method uses a high-resolution scanner, which can be successfully applied to a large number of specimens and offers some advantages over the traditional method, as the equipment required is less expensive and provides a convenient and less complicated procedure for the operator. The resulting images are processed using ImageJ software [19].

### Characterization of Initial Materials

Granulated ferrous slag from a Finland metallurgical plant was used as the aluminosilicate precursor. In the laboratory, the slag was dried at a temperature of 100 °C and ground using a ball mill. The chemical composition of the metallurgical slag is given in Table 1. The chemical composition of slag was evaluated using XRF analysis. For this analysis, a fluorescence spectrometer S8 Tiger (Bruker AXS, Karlsruhe, Germany) operating at the counter gas Helium 2 bar was used. The slag was mostly composed of calcium, silicon and aluminum oxides. Similar chemical composition of slag was determined by Esmaily et al. [11].

**Figure 2:**
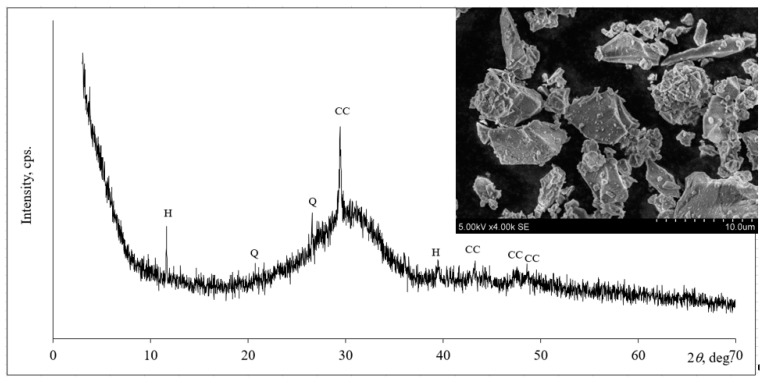
The mineral composition (X-ray diffraction patterns) and microstructure of slag. Notes: CC—calcium carbonate, CaCO_3_ (72–1651); Q—quartz, SiO_2_ (83–539); H—hydrotalcite, Mg_6_Al_2_CO_3_(OH)_16_·4H_2_O (14–191).

Microscopic analysis (Figure 2) showed irregularly shaped, sharp-edged slag particles that varied in the material. X-ray analysis of the slag (Figure 2) showed that the peaks could be assigned to the minerals of calcium carbonate, quartz and hydrotalcite. The initial mineral slag, phosphogypsum and hardened aerated alkali-activated composite were tested according to XRD analysis. A DRON-6 X-ray diffractometer was used to determine the mineral composition. It has Bragg–Brentano geometry using Ni-filtered Cu Kα radiation and a graphite monochromator, which operates at a voltage of 30 kV and an emission current of 20 mA. The step-scan covered an angular range of 2–70° in steps of 2 = 0.02°.

**Figure 3:**
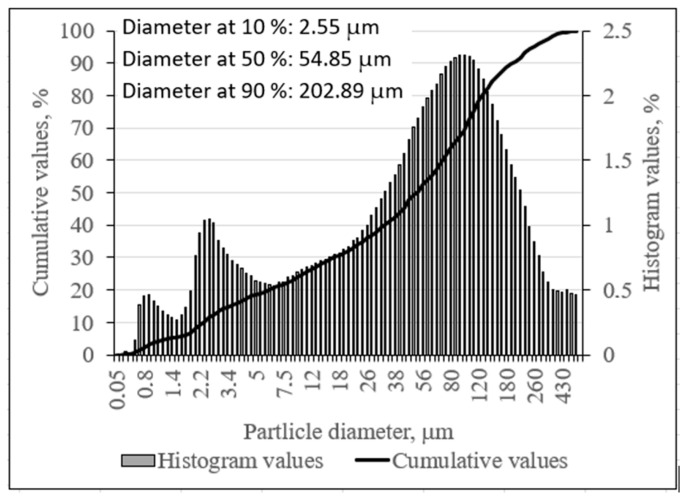
Slag particle size distribution.

The results of granulometric analysis (Figure 3) show that 90% of the slag consisted of particles with a size of 202.89 μm. The specific surface area of the metallurgical slag powder was 207 m^2^/kg, based on the results of the Blaine analysis. The particle size distribution of the slag and phosphogypsum was determined by a laser particle size analyzer (Cilas 1090 LD). The distribution of solid particles in the airstream was 12–15 wt%. Compressed air (2500 mbar) was used as a dispersing phase.

The phosphogypsum used in this study was of the α-hemihydrate type and was obtained from a fertilizer production plant. Phosphogypsum is obtained by extracting phosphoric acid from natural apatite. The resulting powder was dried in the laboratory at 100 ± 5 °C. The chemical composition of the phosphogypsum was determined by the XRF method. This material consisted mainly of calcium and sulfur oxides (Table 2).

**Figure 4:**
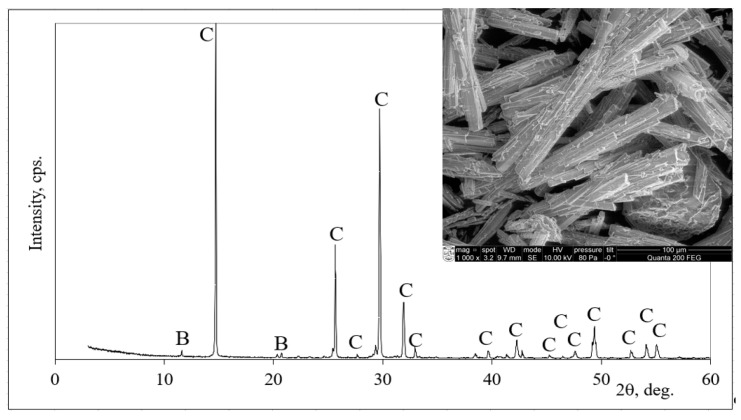
The mineral composition (X-ray analysis) and microstructure of phosphogypsum. Notes: C—basanite, CaSO_4_·0.5H_2_O (33–310); B—brushite, CaPO_3_(OH)·2H_2_O (11–293).

The microstructure of phosphogypsum consisted of irregularly shaped hemihydrate phosphogypsum crystals, as observed in the picture from Figure 4. The scanning electron microscopy (SEM) analysis of the initial material slag, phosphogypsum and hardened porous alkali-activated composite was performed by the high-resolution scanning electron microscope Hitachi S-3400 N, Tokyo, Japan, which guarantees high-resolution images (10 nm at 3 kV, 3 nm at 30 kV).

The X-ray analysis of phosphogypsum (Figure 4) showed peaks belonging to basanite (this mineral dominated) and low-intensity peaks characteristic of brushite (only traces were detected).

**Figure 5:**
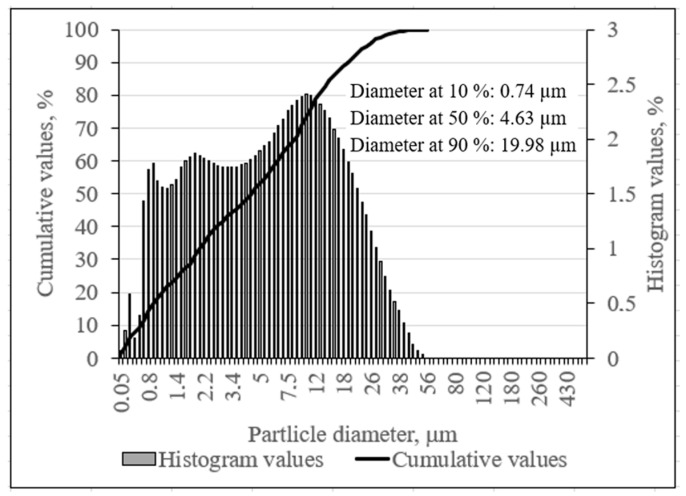
The particle size distribution of phosphogypsum.

Based on the results of granulometric analysis (Figure 5), the phosphogypsum consisted of 90% particles with a size of 19.98 μm. A Blaine^®^ instrument was used to determine the specific surface area of the phosphogypsum powder (201 m^2^/kg).

The alkali activating agent used in this study was sodium hydroxide (NaOH) in granular form (country of origin—Russia). Hydrogen peroxide solution (H_2_O_2_) in a concentration of 35% was used as a blowing agent for alkali-activated slag (country of origin—Poland). The admixture of hydrogen peroxide was 2% and was calculated from the mixture of slag and phosphogypsum. A similar amount of this foaming agent (H_2_O_2_ solution) was added to the alkali-activated pastes of Vaou et al. [10] and Ducman et al. [7].

In this study, dry materials, such as slag and phosphogypsum, were selected according to the principle of rational composition search, in which the amount of one substance was reduced, and that of the other was proportionally increased. The amounts of sodium hydroxide, water and hydrogen peroxide were reduced and increased accordingly until the appropriate amounts of the specific substance were reached.

Five mixtures with different compositions were selected and analyzed (Table 3). The porous specimens were prepared by mixing slag and phosphogypsum powder with sodium hydroxide and hydrogen peroxide solutions.

The manufacturing process of the alkaline-activated material was divided into five steps. Step 1—weighing the amount of raw materials according to the composition. Mixing the solids, dissolving the activator granules in water, and activating the solids. Mixing the mixture and incorporating the foaming agent into the composition. Step 2—pouring and thickening the alkaline-activated mixture. The mixture in the molds was isolated at room temperature for 24 h. Step 3—curing the material in an oven preheated to 60 °C for 24 h. Step 4—formation and isolation of the alkali-activated material over a period of 3 to 28 days, depending on the planned tests. Step 5—at the end of the specified isolation period, the specimens are analyzed using specific test methods.

**Figure 6:**
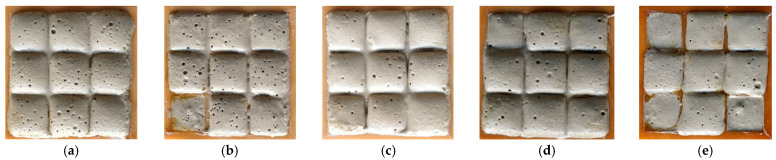
Initial foaming and expansion of the specimen mixture with different phosphogypsum addition: (**a**) P0, (**b**) P3, (**c**) P5, (**d**) P7, (**e**) P10.

In the first step, after the incorporation of the foaming agent into the alkali-activated material mixture, an active reaction was observed—an expansion of the material shown in (Figure 6). In the second step, specimens of the alkali-activated material poured into 20 × 20 × 20 mm silicone molds still showed rapid expansion, which started in the first step and lasted for 10–15 min. Each mixture showed different expansion and reaction characteristics. Increasing the amount of phosphogypsum in the mix composition showed less expansion of the specimens and better gasification behavior during mixing. After the third step, no change in the mixture composition was observed for the P0, P3 and P5 specimens, but a decrease in the porosity of the P7 and P10 specimens was observed. Before adding the alkali-activated material, the molds were greased for easier specimen formation.

The initial aeration of the mixture immediately after molding the specimen showed that specimens P0 without phosphogypsum addition had an early volume expansion of up to 5%. However, there was no initial expansion in the specimens with a higher content of phosphogypsum admixture. This is very convenient for the technological shaping of the products and gives more time to shape the products and adjust their properties by changing the amount of phosphogypsum additive.

**Figure 7:**
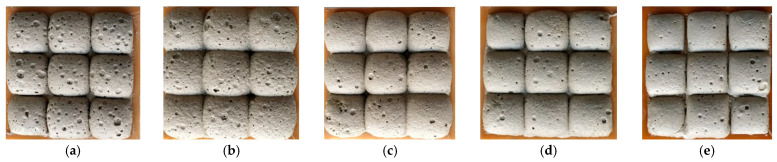
Final foaming and expansion of the specimen mixtures with different phosphogypsum addition: (**a**) P0, (**b**) P3, (**c**) P5, (**d**) P7, (**e**) P10.

The technological principle of preparing the alkali-activated material is shown in a graphical abstract, and the results of material expansion are shown in (Figure 7).

Based on these compositions, the properties of the materials were studied and the dependencies on the phosphogypsum content in the mixture composition were determined.

The final aeration of the mixture was visible after 3 h. The test showed that the samples P0, without the addition of phosphogypsum, showed the greatest increase in volume of up to 25%. In contrast, the samples with the phosphogypsum addition P10 had a volume increase of up to 15%.

## 3. Results and Discussion

**Figure 8:**
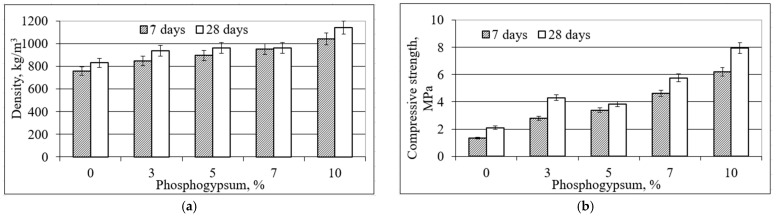
Dependence of density (**a**) and compressive strength (**b**) of porous alkali-activated specimens on the amount of phosphogypsum used in the mixture after hardening for 7 and 28 days.

The addition of phosphogypsum had a significant effect on the density and compressive strength of porous alkali-activated specimens. The density increased by increasing the amount of phosphogypsum in the system, as can be seen in Figure 8a. The similar values of density were after 7 and 28 days of hydration. After 7 days of hydration, the values of density changed from 759 kg/m^3^ to 1040 kg/m^3^ without phosphogypsum and by the incorporation of 10% phosphogypsum, respectively. After a longer duration of hydration (28 days), the values of density slightly increased compared with the density after 7 days. It changed from 830 kg/m^3^ to 1142 kg/m^3^ for the specimens without phosphogypsum and with 10% phosphogypsum, respectively. According to Şahin et al. [8], a similar density (560–750 kg/m^3^) was achieved for porous alkali-activated slag that was cured at a temperature of 80 °C.

The density of the specimens was closely related to the compressive strength values. The compressive strength properties are closely related to the phosphogypsum content in the composition. The compressive strength of the samples was tested using a hydraulic press (ToniTechnik 2020). At least three samples were tested for each type. The density of the porous composite was determined according to EN 12390-7, and the compressive strength of samples was determined according to EN 196-1. The higher amount of phosphogypsum in the system caused a faster hydration process, so a higher strength was achievable [5,20]. By increasing the phosphogypsum from 0 to 10%, the compressive strength of the specimens varied in the range of 1.35–6.21 MPa after 7 days and increased up to 2.11–7.95 MPa after 28 days (Figure 8b) depending on the phosphogypsum content. Luna-Galiano et al. [21] stated that the compressive strength after 28 days was related to the porosity values and density of the specimens. The porous specimens based on coal combustion fly ash had compressive strength in the range of 4.2–5.32 MPa, and the density was in the range of 916–1238 kg/m^3^, as reported by Leiva et al. [22]. Dembovska et al. [23] prepared lightweight material with a density of 380–470 kg/m^3^ with 1.1 and 2 MPa of compressive strength. As can be seen, the results obtained in the present work are in the same range as the geopolymers described in those studies.

**Figure 9:**
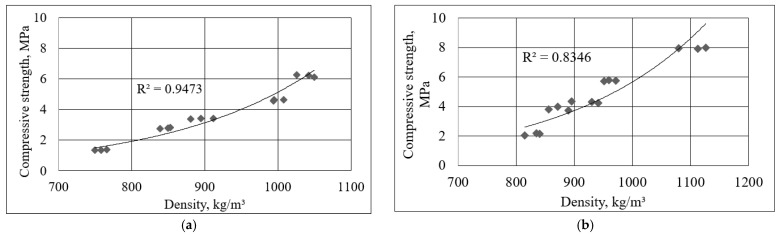
Relationship between density and compressive strength after 7 days (**a**) and 28 days (**b**) of hardening at different additions of phosphogypsum.

The relationship between the density of the porous alkali-activated slag and its compressive strength is shown in Figure 9. A strong exponential correlation was found between compressive strength and density. The coefficient R^2^ was 0.9473 for the specimens after 7 days of hydration and became slightly lower after 28 days (R^2^ = 0.8346). The lowest density was 815 kg/m^3^, with a compressive strength of about 2.12 MPa. The compressive strength increased up to 7.95 MPa when the density was 1080 kg/m^3^.

**Figure 10:**
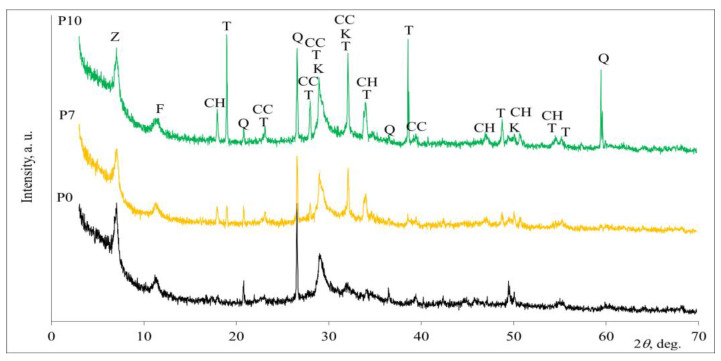
X-ray diffraction patterns of porous alkali-activated slag. Notes: Z—sodium aluminum silicate hydrate, Na_96_Al_96_Si_96_O_384_216H_2_O (39-222); K—calcium silicate hydrate, Ca_1.5_SiO_3.5_H_2_O (33–306); Q—quartz, SiO_2_ (83–539); T—thenardite, Na_2_SO_4_ (74–2036); CC—calcite, CaCO_3_ (72–1937); F—hydrotalcite, Mg_6_Al_2_CO_3_(OH)_16_·4H_2_O (14–191); CH—portlandite, Ca(OH)_2_ (84–1268).

Specimens with three different compositions (Table 3) were subjected to XRD analysis after 28 days of curing. Based on the X-ray diffraction patterns (Figure 10), the predominant peaks belonging to the minerals of quartz, hydrotalcite and calcite were found in the specimens containing 0, 7 and 10% phosphogypsum. These three minerals remained unreacted after the alkaline activation process, but new compounds were formed as well.

In the alkali environment, the dissolved CaSO_4_·2H_2_O from the phosphogypsum reacted with sodium hydroxide and led to the formation of specific reaction products: thenardite and portlandite. The content of portlandite and thenardite gradually increased in the presence of 7% and 10% phosphogypsum. Equation (1) shows the reaction of phosphogypsum and sodium hydroxide:

CaSO_4_·2H_2_O + 2NaOH → Na_2_SO_4_ + Ca(OH)_2_ + 2H_2_O (1)

Thenardite formed as alkaline activation increased the pH of the mixture, accelerated slag dissolution, and a reaction occurred between portlandite and slag. It can be concluded that the incorporation of phosphogypsum in the composition of alkaline-activated slag affects the formation of C-S-H, CaCO_3_ and Na_2_SO_4_ minerals, which correlate with the determined values of compressive strength. Rashad et al. [24] stated that a small amount of Na_2_SO_4_ led to the better compactness of the alkali-activated slag matrix, which had a significant effect on its mechanical properties. The material produced under these conditions showed similar mineral composition to the non-porous alkali-activated slag made without the porous creating agent [5,24].

**Figure 11:**
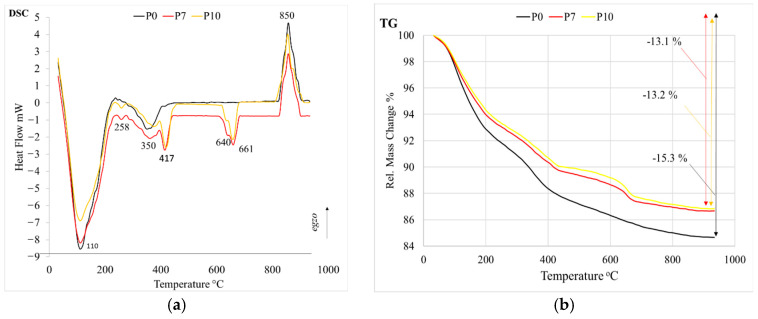
Differential scanning calorimetry (**a**) and thermogravimetric analysis (**b**) of porous alkali-activated slag at 28 days.

The results of the differential scanning calorimetry are in good agreement with the results of the XRD analysis (Figure 11).

All DSC curves of the porous alkali-activated slag show endothermic peaks at 110–113 °C, which are attributed to the release of absorbed water in the capillary pores and the dehydration of the C-(A)-S-H gel phase (Figure 11a) [25]. The small DSC peaks centered at about 258 °C are the result of the decomposition of thenardite (Na_2_SO_4_) [26]. These small endothermic peaks are observed in the specimen with phosphogypsum (P7 and P10). The endothermic peaks at about 350 °C and 375 °C are related to the dehydration of sodium aluminosilicate hydrate [27]. The endothermic peaks in the temperature range of 417–418 °C are due to the dehydration of Ca(OH)_2_ [28]. These peaks are also only found in the samples with phosphogypsum addition. The endothermic DSC peaks centered at about 640 °C are the result of the dehydration of calcium silicate hydrate, and the decarburization of calcite is attributed to the endothermic peak at about 661 °C [29,30]. In addition to the endothermic peaks, the exothermic peaks at about 850 °C, which were detected in all samples, could be attributed to the transformation of C-(A)-S-H into wollastonite (CaSiO_3_) [31]. The total mass loss of 15.27% was higher in the P0 samples without phosphogypsum than in the samples P7 and P10 with phosphogypsum (13.24% and 13.13%, respectively) (Figure 11b).

A variety of materials are being researched, including highly porous materials, which are driving innovation in the production of porous alkali-activated materials. Alkali ash activation is very different from the hydration process of Portland cement but also has similarities with zeolite synthesis. The type of activator, pH and mineral content of the mixture have a direct influence on the mechanical properties, porosity and activation process of the alkali-activated material.

**Figure 12:**
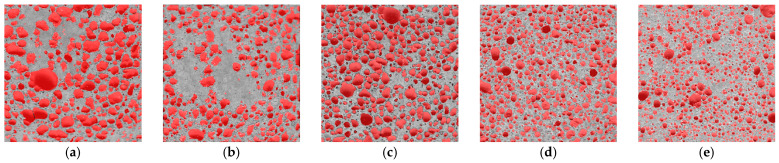
Pores and their distribution shown in specimen cross-sectional cuts in the hardened material according to the mixture: (**a**) P0, (**b**) P3, (**c**) P5, (**d**) P7, (**e**) P10.

The physical and mechanical properties of a porous material are highly dependent on the distribution of pores in the material in different sizes. Materials with the same total porosity can have completely different properties due to the small number of large pores or the large number of small pores. The structure of porous materials can be extracted in two ways: mechanically and chemically. In the mechanical method, the paste is physically mixed with the prepared foam. In the chemical method, oxygen reacts with the added materials and releases gas. Gas evolution plays a key role in forming the structure of the porous material. The curing conditions have a significant influence on the formation of micro and macro structures of alkaline-activated ash. The images of specimen P0 (Figure 12) show a distribution of irregularly shaped air voids. Then specimen P10 shows a dense and uniform cross-sectional distribution of fine pores and regularly oval-shaped air voids.

**Figure 13:**
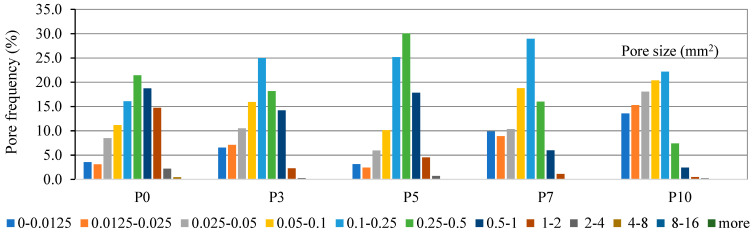
Pore system characterization of specimens.

The air voids of sample P0 of the mixture composition ranged from 0.0125 to 8 mm^2^, of which 21.4% were voids larger than 0.5 mm^2^. The determined area of the air voids in the cross-section was 47.5%. In the P10 sample mixture, the size of the air voids predominates between 0.0125 and 4 mm^2^, with the largest proportion, 22.2%, consisting of 0.25 mm^2^ pores (Figure 13). The cross-sectional area of the air voids was determined to be 36.2%.

Examination of the samples of all compositions shows that the higher the content of phosphogypsum admixture in the mixture, i.e., the higher the number of pores visible in the section, but the smaller their size, the more favorable the conditions for void and pore formation. In this way, the structure of the mixture is more compact, and the mechanical properties are better. These conditions change successively as the composition of the mixture changes.

D. Xuan’ et al. determined the cross-sectional porosity of the alkali-activated ash material. Depending on the ratio of ash to glass powder, the determined values ranged from 23.3% to 42.2%. [32]. The porosity values determined in the final work were mostly in the range of 35.7–47.5%, which is close to the porosity values reported in the scientific literature. Specimens with higher porosity were characterized by larger pores and a denser cross-sectional distribution.

Thermal conductivity describes the property of a material to transfer thermal energy between adjacent molecules. This is one of the most important properties of the insulating material, which is evaluated according to the value of the coefficient λ (W/m·K), obtained according to the methodology of the standard LST EN 12667:2002 [33].

During the research work, the coefficients of thermal conductivity λ (W/m·K) of specimens of various alkaline-activated material compositions were determined. Selected compositions of mixtures of specimens: P0, P5 and P10 with different amounts of phosphogypsum in the mixture, i.e., 0, 5 and 10%, respectively. The external dimensions of the specimens were 300 × 300 × 50 mm, selected according to the requirements of the standard LST EN 12667:2002 [33].

To avoid the influence of humidity on the test results, the specimens were dried in an oven at 60 °C for 24 h before determining the thermal conductivity. The obtained thermal conductivity values were mainly in the range of 0.0985–0.2618 W/m·K. An increase in thermal conductivity with increasing phosphogypsum content was observed. The porous alkali-activated material containing no phosphogypsum had the best thermal conductivity properties, i.e., λ = 0.0985 W/m·K. In formulations containing 5% and 10% phosphogypsum, the thermal conductivity increased by 88.1% and 165.8%, respectively.

According to the research results of J. Henon’o, A. Alzina et al., the thermal conductivity of the porous material of alkaline-activated ash ranged from 0.12 to 0.33 W/m·K [34]. Z. Zhang’, J.L. Provis, et al. stated that porous alkali-activated fly ash could have a thermal conductivity in the range of 0.22 to 0.24 W/m·K [35]. The values of the coefficient λ (W/m·K) obtained in the final work were between 0.0985 and 0.2618 W/m·K, which is close to the values reported in the literature.

**Figure 14:**
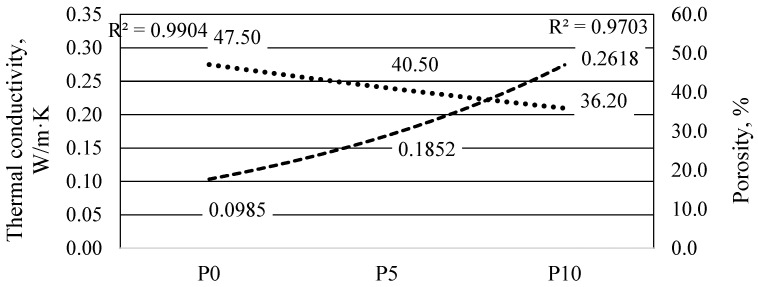
Dependence of the thermal conductivity coefficient and pore percentage of the material on the amount of phosphogypsum in the material mixture.

Based on the thermal conductivity and air void distribution data of the specimens shown in Figure 14, it can be concluded that the number of air voids has a direct effect on the thermal conductivity coefficient. The specimen without phosphogypsum had the largest air void size (0.0125–8 mm^2^) and cross-sectional area (47.5%) and the lowest thermal conductivity value (λ = 0.0985 W/m·K). The specimens mixed with 5% and 10% phosphogypsum had smaller pores and cross-sectional areas and higher thermal conductivity. It can be concluded that the values of the thermal conductivity coefficient λ (W/m·K) are directly dependent on the pore size and its distribution in the cross-section of the specimen.

Compared to the construction market of the countries of the Baltic Sea region, the properties of the material produced with the presented products are comparable to those of aerated concrete. Of course, aerated concrete products differ according to their production technology or the type of fillers. Aerated concrete is most commonly used for low-rise residential construction, or more precisely, building blocks made of this type of concrete with a density of 400–600 kg/m^3^, a compressive strength of 2.0–5.0 MPa, and a thermal conductivity coefficient of 0.09–0.12 W/m·K. The porous material of alkali-activated slag had high early compressive strength after 7 days of curing as it reached 64–88% of its ultimate strength. By increasing the phosphogypsum from 0 to 10%, the compressive strength of the specimens varied in the range of 1.35–6.21 MPa after 7 days and increased up to 2.11–7.95 MPa after 28 days, depending on the phosphogypsum content. The thermal conductivity of the alkali-activated slag porous material was determined to be λ = 0.0985 W/m·K, and the thermal conductivity of phosphogypsum increased it by 88.1% (λ = 0.1852 W/m·K) after adding 5% to the material, the addition of 10% increased the thermal conductivity of phosphogypsum by 165.8% (λ = 0.2618 W/m·K). If one compares the properties of the products, it can be seen that material properties with similar or even better parameters than those of ordinary aerated concrete were achieved using metallurgical slag during this study.

## 4. Conclusions

- The recommended content of phosphogypsum in alkali-activated material is 3–5% due to the optimal distribution of the mechanical and thermal properties and the conductivity. Alkali-activated slag and phosphogypsum material can be used in the manufacture of low-strength insulation blocks and to protect structures from the effects of high temperatures.
  The porous material of alkaline-activated slag had relatively high early compressive strength properties after 7 days of curing as it reached 64–88% of its ultimate strength. By increasing the phosphogypsum from 0 to 10%, the compressive strength of the specimens varied in the range of 1.35–6.21 MPa after 7 days and increased to 2.11–7.95 MPa after 28 days (Figure 8b), depending on the phosphogypsum content. A direct relationship between the density and compressive strength of the AAM was found when 3–10% phosphogypsum was added.
  The values of porosity of the porous alkali-activated slag in the cross-section ranged from 35.7 to 47.5%. Specimens with 5% phosphogypsum had 14.7% lower porosity, and specimens with 10% phosphogypsum had 23.8% lower porosity compared to the control without phosphogypsum.
  The thermal conductivity of alkali-activated slag porous material was determined to be λ = 0.0985 W/m·K, and the thermal conductivity of phosphogypsum increased by 88.1% (λ = 0.1852 W/m·K) after adding 5% to the material, the addition of 10% increased the thermal conductivity of phosphogypsum by 165.8% (λ = 0.2618 W /m·K). The coefficient of thermal conductivity λ (W/m·K) directly depends on the pore size and distribution in the specimen cross-section. It can be concluded that phosphogypsum has an effect on density and porosity and the thermal conductivity properties of the alkali-activated porous material.
  This study found that the absence of phosphogypsum in the composition of the specimen mixes or the incorporation of a small amount of it showed good thermal conductivity properties, but the bonding conditions, expansion, density and compressive strength of the specimen were inadequate. This would explain the annealing of phosphogypsum, even in small quantities.

## Figures and Tables

**Table 1:** The XRF analysis showing the chemical composition of the slag, wt%.

SiO_2_	CaO	Al_2_O_3_	Fe_2_O3	MgO	K_2_O	P_2_O_5_	SO_3_	BaO	SrO	TiO2	Na_2_O	Other
37.7	45.2	6.44	0.79	5.96	0.52	0.068	1.85	0.068	0.069	0.29	1.02	0.025

**Table 2:** The chemical composition (based on XRF analysis) of hemihydrate phosphogypsum from Kovdor, wt%.

SiO_2_	CaO	Al_2_O_3_	Fe_2_O3	MgO	K_2_O	P_2_O_5_	SO_3_	F	SrO	TiO2	Na_2_O	Other	L.I. *
0.34	39.06	0.07	0.04	0.21	-	1.61	52.21	0.06	-	-	-	-	6.4

* L.I.—the loss on ignition was determined at 400 °C.

**Table 3:** The composition of the mixtures and the use of initial materials, wt%.

Mix	Slag, %	Phosphogypsum, %	NaOH, %	H_2_O, %	H_2_O_2_, %
P0	100	0	9.71	27.0	2.0
P3	97	3	9.71	27.0	2.0
P5	95	5	9.71	27.0	2.0
P7	93	7	9.71	27.0	2.0
P10	90	10	9.71	27.0	2.0
